# Mass Spectrometry-Based Omics: An Integrated Framework for Analytical Quality, Data Integrity, and Biological Interpretation

**DOI:** 10.3390/molecules31203604

**Published:** 2026-10-10

**Authors:** Andrzej Gawor, Ewa Bulska

**Affiliations:** University of Warsaw, Faculty of Chemistry, Biological and Chemical Research Centre, Żwirki i Wigury 101, 02-089 Warsaw, Poland

**Keywords:** mass spectrometry, omics, quality control, quality assurance, data release, system suitability, fit-for-purpose, traceability, artificial intelligence, claim strength

## Abstract

Results obtained with mass spectrometry-based omics depend on every stage of the work, from sample collection to data processing. Quality guidance exists for individual omics fields and for individual stages, but it rarely states when a dataset is ready for statistical analysis or how far its interpretation may go. We present a step-by-step protocol for proteomics, metabolomics, lipidomics, glycomics/glycoproteomics, and metallomics that brings existing recommendations and international standards together in six steps: (1) defining the intended use and the target claim, (2) checking that each specimen is fit for analysis, (3) preparing samples and acquiring data under control, (4) checking the dataset before release, (5) analysing and interpreting the data, including the use of tools based on artificial intelligence, and (6) verifying the findings and reporting. For each step, the protocol lists the required input, the actions to be taken, a checkpoint with acceptance criteria set in advance, the corrective action to be taken when the criteria are not met, and the record to be kept. Five claim levels show which conclusions the collected evidence supports. Separate criteria are given for qualitative, semi-quantitative, and quantitative studies, and numerical values from the literature are offered as starting points that each laboratory should replace with its own limits. The protocol can be used as a practical guide for planning, carrying out, and reporting omics studies and for deciding when a finding needs further verification.

## 1. Introduction

Mass spectrometry (MS) is used to measure proteins and proteoforms [1], metabolites [2], lipids [3], glycans and glycoconjugates [4], and elemental species [5,6]. Analytical depth alone does not make such data reliable. Sample handling before analysis can change the molecular profile [7,8]. Instrument drift and batch effects can imitate or hide biological differences [9,10]. Laboratories analysing the same sample have reported different results, mainly because of differences in database searching and data processing [11,12]. Data-independent acquisition (DIA) [13,14], ion mobility [15], low-input and single-cell methods [16], MS imaging [17], and spectrum prediction based on artificial intelligence (AI) [18] have extended coverage and throughput. At the same time, they have added many settings that can change the final result. The quality of an omics result therefore has to be demonstrated for the whole chain of work, from the specimen to the interpretation.

Several community initiatives have published guidance for individual fields. Examples are the Minimum Information About a Proteomics Experiment (MIAPE) [19], the identification levels of the Metabolomics Standards Initiative (MSI) [20], quality assurance/quality control (QA/QC) recommendations for untargeted metabolomics [9,21], good-practice recommendations and reporting checklists for lipidomics [3,22], the Minimum Information Required for a Glycomics Experiment (MIRAGE) [4,23], the mzQC format for exchanging QC metrics [24], and international standards for biobanks, testing laboratories, and medical laboratories [25,26,27]. These documents are essential, yet they are difficult to use as one working procedure. Each covers a single field or a single stage of the work, whereas in practice sample management, measurement, data processing, and interpretation are often carried out by different teams. Most of them describe what should be reported. Few say what should happen when a criterion is not met, at which point the decision should be made, or whether a failure calls for new measurements, reprocessing, or a more cautious conclusion. They also do not link the analytical evidence to the strength of the conclusion. Surveys and interlaboratory studies show that QC practice and acceptance limits differ widely between laboratories [28,29], and the slow progress of biomarkers from discovery to clinical use has been attributed in part to conclusions that went beyond the evidence [30].

Quality terms are also used differently in different fields. A QC sample may be a system suitability mixture, a pooled study sample, a characterised digest, an isotope-labelled internal standard, or a certified reference material, and each of them can only report on the stages it has passed through [9,31]. In the same way, a numerical limit that works in one setting, such as a false discovery rate of 1% for peptide identification or a relative standard deviation (RSD) of 20–30% for features in pooled QC samples, cannot simply be transferred to another matrix, platform, or type of quantitative study [9,32].

**Figure 1:**
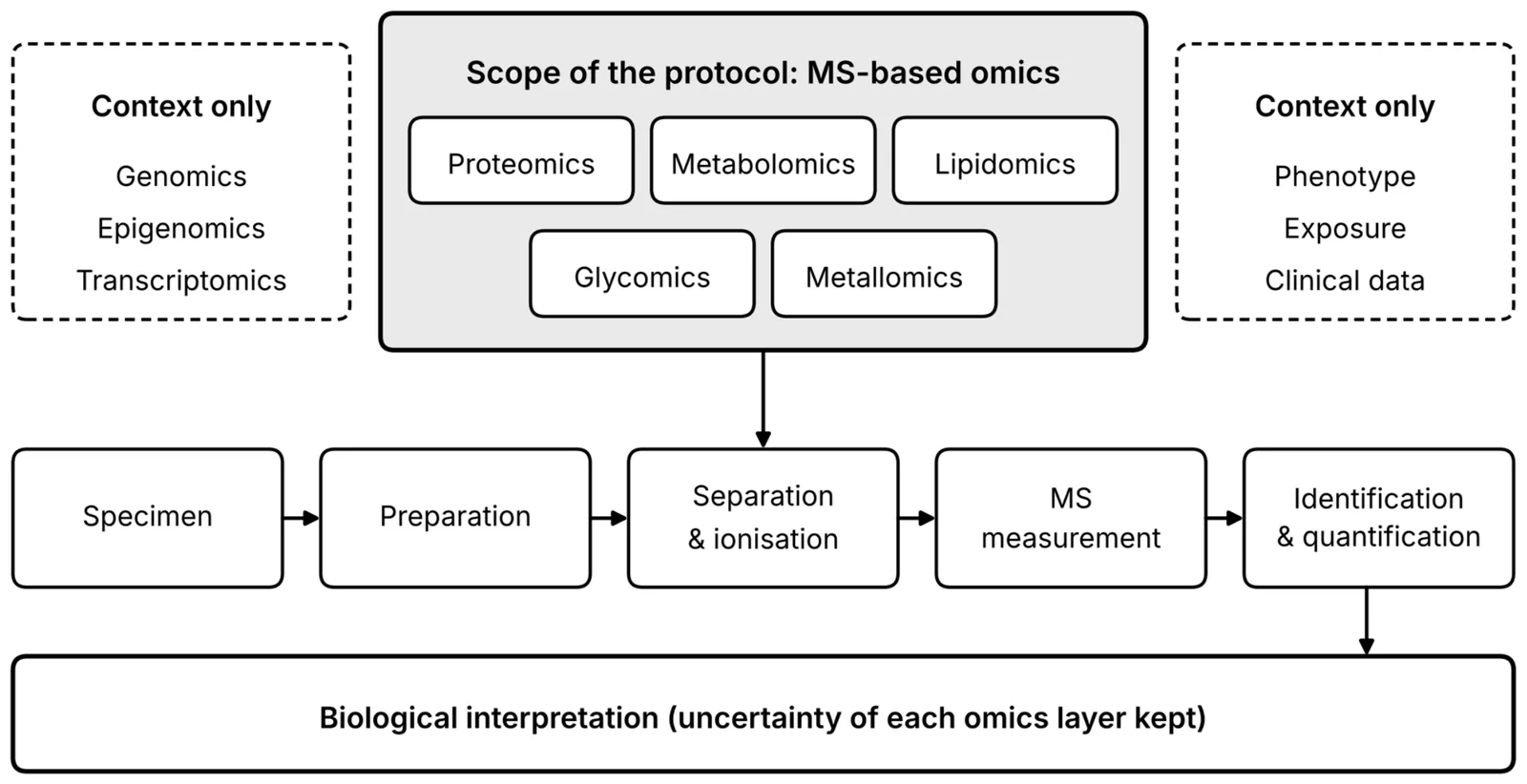
Scope of the protocol. The protocol covers MS-based proteomics/proteoforms, metabolomics, lipidomics, glycomics/glycoproteomics, and metallomics/speciation (grey box). Genomic, epigenomic, and transcriptomic data, as well as phenotype, exposure, and clinical data, are used only as context during data integration (dashed boxes). All MS-based fields share the same analytical sequence (middle row), and the uncertainty of each field is kept when the results are combined (bottom).

The aim of this work is to combine the existing guidance into one practical protocol that can be followed step by step. The protocol consists of six steps, from defining the intended use to verifying the findings. Each step is described in the same way: required input, actions, a checkpoint with criteria fixed in advance, the corrective action when the checkpoint is not passed, and the record to be kept. Five claim levels connect the evidence collected in the six steps with the conclusions that may be drawn. The protocol also specifies how a failure should be corrected depending on where it arose, and it gives separate quality criteria for qualitative, semi-quantitative, and quantitative studies. It does not replace field-specific guidance or the validation of individual assays; it shows where these documents apply and what follows from their results. The protocol covers omics fields in which MS is the main measurement technique. Genomic, epigenomic, and transcriptomic data enter only as context during data integration (Figure 1) [33,34,35]. Section 2 describes how the protocol was developed and which of its parts were adopted, adapted, or newly proposed.

## 2. Materials and Methods: Development of the Protocol

### 2.1. Design Approach

We developed the protocol in two parts. First, we carried out a structured narrative review of peer-reviewed literature, international standards, and community guidance on quality in MS-based proteomics, metabolomics, lipidomics, glycomics/glycoproteomics, and metallomics. Second, we converted the recommendations collected in the review into the actions and checkpoints of the six steps. The work had four stages: finding sources (Section 2.2), selecting them (Section 2.3), extracting and comparing recommendations across omics fields (Section 2.4), and incorporating them into the protocol according to predefined rules (Section 2.5). No new biological material, experimental data, or patient data were generated, so ethical approval and informed consent were not required. The review was narrative, not systematic, and no meta-analysis was performed.

### 2.2. Identification of Sources

The literature was searched up to 12 September 2026 in PubMed and on publisher and standards websites, and the reference lists of consensus documents and benchmark studies were followed backwards and forwards. Search terms included ‘mass spectrometry’, ‘omics’, ‘quality control’, ‘quality assurance’, ‘system suitability’, ‘preanalytical’, ‘biobanking’, ‘metabolomics QC’, ‘lipidomics standards’, ‘MIRAGE’, ‘proteomics QC’, ‘mzQC’, ‘interlaboratory’, ‘normalisation’, ‘artificial intelligence’, ‘DIA’, ‘spectral prediction’, ‘missing values’, ‘verification’, ‘sample size’, ‘power’, and ‘multi-omics integration’. Bibliographic data and DOIs were checked against publisher and database records. The status of each standard and guideline was checked in the catalogues of the International Organization for Standardization (ISO) and the International Council for Harmonisation (ICH).

### 2.3. Eligibility and Selection

We included five types of sources: (A) international standards and harmonised guidelines on biobanking, laboratory competence, development and validation of analytical procedures, bioanalytical method validation, and medical laboratory examinations [25,26,27,36,37,38]; (B) consensus recommendations and reporting standards of recognised initiatives, such as the Proteomics Standards Initiative, the Metabolomics Standards Initiative, the Metabolomics Quality Assurance and Quality Control Consortium (mQACC), the Lipidomics Standards Initiative, MIRAGE, and the International Society for Biological and Environmental Repositories (ISBER) [7,19,20,21,22,24,39]; (C) multi-laboratory, interlaboratory, and benchmarking studies that measured sources of variation [11,29,40,41,42,43]; (D) methodological papers describing QC metrics, software, statistical methods, or AI models in current use [44,45,46,47,48]; and (E) original papers that introduced concepts still in use, such as MIAPE, MSI identification levels, target–decoy estimation of the false discovery rate, the FAIR principles, and fit-for-purpose targeted MS assays [19,20,32,49,50]. We excluded single application studies without general quality content, guidance on non-MS techniques (except where needed to define the scope of data integration), and non-peer-reviewed sources other than standards, guidelines, and official repository documentation. Where a guideline existed in several versions, we used the most recent one. Older versions were kept only when current guidance still refers to them, as is the case for the MIRAGE sample preparation guidelines [23]. Our own methodological studies were included only where they deal directly with quality control in liquid chromatography–high-resolution MS (LC–HRMS) or with the evaluation of missing values [51,52].

### 2.4. Extraction and Comparison Across Omics Fields

For each source, we recorded (i) the stage of work addressed (planning, specimen, preparation, acquisition, processing, interpretation, verification, or reporting), (ii) the object of quality control (specimen, procedure, instrument, data, model, or conclusion), (iii) the type of recommendation (reporting requirement, procedural control, acceptance criterion, numerical limit, or corrective action), (iv) the omics field, and (v) the type of evidence (standard, consensus document, interlaboratory or benchmarking study, or single-laboratory study). The recommendations were then compared across omics fields, stage by stage. This comparison showed which recommendations are shared by all fields, which depend on the chemistry or on the measured quantity of a particular field, and at which stages guidance is missing, inconsistent, or limited to reporting. Its results are summarised in the tables accompanying the protocol.

### 2.5. Rules for Incorporation and Definition of Terms

Five rules were applied. Rule 1: a recommendation became a universal principle when it concerned an object of quality control common to all MS-based omics fields and was supported by an international standard or by guidance from at least two fields. Rule 2: a recommendation was kept as a field-specific control when its rationale depended on the chemistry or measured quantity of one field, for example, class-specific internal standards in lipidomics or the preservation of chemical species in metallomics. Rule 3: numerical values were included only when they had been proposed in community guidance or were widely used in benchmark studies; each value is cited and labelled as a starting point. Rule 4: where guidance was missing or inconsistent, the protocol gives the decision rule and asks for limits derived in the laboratory, instead of transferring limits from better-studied fields. Rule 5: each control was placed at the earliest step at which its failure can be detected, and each corrective action at the step where the failure arises.

Two terms used in this protocol require a definition. Universal principles are requirements that apply to every MS-based omics field, regardless of platform or matrix: acceptance criteria defined in advance, controls added before the stage they are meant to monitor, balanced allocation of samples to batches, retention of raw data, version control of data processing, and independent verification when the claim requires it. Illustrative operational starting points are numerical values proposed in community guidance or widely used in benchmark studies, such as an identification false discovery rate of 1% in proteomics [32,53] or an RSD limit of 20–30% for features in pooled QC samples in untargeted metabolomics [9,21]. They are intended as initial values for method development and should be confirmed or replaced by limits derived in the laboratory. Both terms were introduced for this protocol. They apply in practice the fit-for-purpose principle formulated for targeted MS assays [50] and for QA/QC in metabolomics [9,21].

### 2.6. Conceptual Basis and Origin of the Protocol Components

The idea of a sequence of steps, each ending with a documented decision against criteria set in advance, comes from stage-gate models of project management [54] and from the requirements of ISO/IEC 17025:2017 and ISO 15189:2022 to ensure the validity of results and to control nonconforming work [26,27]. The view that performance criteria are defined before method development and monitored during routine use follows ICH Q14 and Q2(R2) [36,37]. The claim levels were adapted from phase models of biomarker development [30,55] and from the tiers of targeted MS assays [50]. Table 1 shows, for each component of the protocol, whether it was adopted without change, adapted, or newly proposed. The steps reflect our experience with laboratory quality systems based on ISO/IEC 17025 and ISO 15189 [26,27] and were checked against our earlier LC–HRMS protocol for assuring the validity of proteomics results [52]. No multicentre or formal consensus validation was carried out (see Section 4.6).

## 3. Protocol: Step-by-Step Guide

### 3.1. How to Use the Protocol

**Figure 2:**
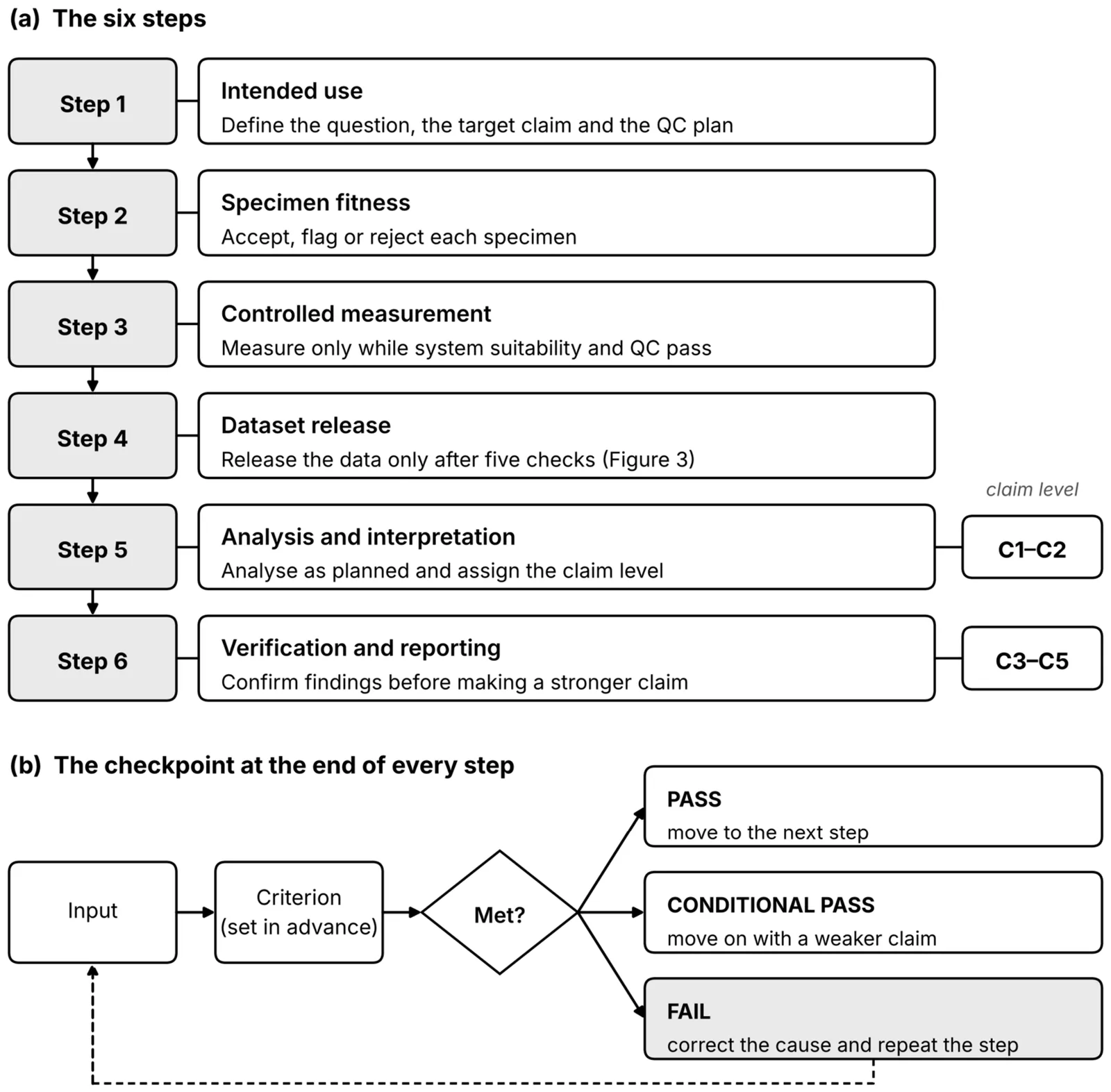
The six steps of the protocol. (**a**) Step 1 defines the research question, the target claim level, and the QC plan before any specimen is processed. Step 2 accepts, flags, or rejects each specimen on the basis of its pre-analytical record. Step 3 allows data acquisition only while system suitability and in-run QC stay within predefined limits. Step 4 releases the dataset only after the five checks shown in Figure 3. Step 5 analyses the released data according to the prespecified plan and assigns the claim level reached. Step 6 confirms the findings in independent material or with orthogonal methods before a stronger claim is made. Claim levels C1–C2 can be reached after Step 5; levels C3–C5 require Step 6 (Table 3). (**b**) The checkpoint at the end of every step. The input is compared with a criterion set in advance. If the criterion is met, the work moves on (PASS). If it is not fully met but the limitation is documented and limited, the work moves on with a weaker claim (CONDITIONAL PASS). Otherwise, the cause is corrected, and the step is repeated (FAIL; dashed arrow). ISO 20387:2018 [25] applies mainly to Step 2 and ISO/IEC 17025:2017 [26] to Steps 3 and 4; ISO 15189:2022 [27] applies directly when the work is carried out in a medical laboratory for clinical use (level C5). QC, quality control.

The protocol consists of six steps carried out in a fixed order (Figure 2, Table 2). Each step is described under the same five headings.

*Input.* What must be available before the step starts.

*Actions.* What to do, in numbered order.

*Checkpoint.* The criteria are fixed before the data are interpreted, and the decision is made against them.

*If the checkpoint is not passed.* The corrective action.

*Record.* What is documented and passed on to the next step.

Three decisions are possible at a checkpoint. PASS means that all criteria are met and the work moves to the next step. CONDITIONAL PASS means that a criterion is not fully met, but the deviation is documented, its effect is limited, and the conclusion is restricted accordingly. FAIL means that the work does not move on until the cause has been corrected.

How a failure is corrected depends on where it arose. Problems in data generation, such as loss of system suitability, drift that cannot be corrected, contamination, or incorrect assignment of files to samples, cannot be repaired by data processing. The affected interval must be reinjected, re-prepared, or reacquired. Problems in data processing, such as an unsuitable database, alignment, or normalisation setting, can be corrected by reprocessing the stored raw data, provided that the original output and the reason for the change are kept. A limitation that cannot be removed but whose effect is limited, for example, a documented pre-analytical deviation in a few specimens, can be accepted as a CONDITIONAL PASS with a restricted conclusion. A claim intended for clinical use (level C5) needs analytical and clinical validation that an exploratory publication (level C1) does not need, including an independent clinical cohort and a validated examination procedure.

Each step has a named person responsible for it, for example, the study leader for Steps 1 and 6, the biobank or sample manager for Step 2, the analytical leader for Step 3, the data analyst for Step 4, and the statistician for Step 5. For conclusions at level C3 or higher, the decisions at Steps 4 and 6 should be reviewed by someone who did not generate the data. The amount of documentation depends on the setting. In research laboratories, a dated, version-controlled entry in an electronic laboratory notebook or a laboratory information management system is sufficient. Laboratories working under ISO/IEC 17025, ISO 15189, good laboratory practice (GLP), or good manufacturing practice (GMP) follow the nonconformity, deviation, and corrective action procedures of their quality system [26,27,66]. Together, the records of the six checkpoints form the release record of the study (Table 2, last column).

### 3.2. Step 1: Define the Intended Use and the Target Claim

*Input.* The research question, the matrix, the study population or model, and the omics field(s). Step 1 is completed before any specimen is processed.

*Action 1.1. Write the intended-purpose statement.* State the biological question, matrix, population or model, omics field(s), main comparison, target claim level (Table 3), and quantitative level (Table 4). This statement guides all later decisions on sample numbers, randomisation, quantitative strategy, QC materials, identification confidence, and verification.

*Action 1.2. Plan biological replication and sample size.* Biological replicates, not repeated injections of one extract, decide whether a biological difference can be generalised. Technical replicates estimate parts of the analytical variation but cannot make up for too few independent samples. When the biological variance or the expected effect size is unknown, run a representative pilot study to estimate the variance and to calculate the sample size or power [48,67]. Treat estimates from very small pilots with caution, and do not use pilot data that served to set thresholds, select features, or tune models as validation data.

*Action 1.3. Randomise, block, and blind.* Distribute samples from the compared groups randomly or in blocks across collection days, sample preparation batches (for example, a 96-well plate or a set of samples extracted together), operators, analytical methods and column lots, and acquisition batches, so that analytical drift is not confounded with biological condition [9,48,56]. Blocking means grouping samples into sets that are processed together, for example, a balanced number of cases and controls, or both members of a matched or longitudinal pair, in one preparation and acquisition batch. Variation between batches is then removed from the comparison of interest instead of being added to it [48]. Use blinding when manual peak review, exclusion of samples, or assessment of endpoints could be influenced by knowledge of group membership.

*Action 1.4. Choose the quantitative level and the matching QC requirements.* Table 4 lists the requirements for each level. Qualitative studies (identification, presence or absence, profiling without quantitative comparison) must control identification confidence and contamination but do not need analyte calibration. Semi-quantitative studies compare relative signal intensities between groups. They need internal standards or normalisation that correct for preparation and injection variability, as well as precision filtering based on pooled QC samples [9,21,68]. Quantitative studies report concentrations or amounts. They need calibration with authentic standards, preferably stable-isotope-labelled internal standards, and accuracy and precision checked against predefined criteria, for which bioanalytical guidelines and the tiers of targeted MS assays give reference values [38,50]. In lipidomics, quantification depends on the lipid class and requires class-matched internal standards [3].

*Action 1.5. Write the statistical analysis plan and the QC plan.* Define endpoints and acceptance criteria before looking at group separation or candidate features, because changing the objective afterwards increases the risk of selective reporting and overfitting. In the QC plan, list each control, the point at which it is added, and the property it monitors (Step 3).

*Checkpoint 1.* Move on only if the intended-purpose statement, the target claim and quantitative levels, the endpoints and acceptance criteria, the sample size justification, the randomisation, blocking, and blinding plan, the statistical analysis plan, and the QC plan are documented and approved.

*If the checkpoint is not passed.* Do not start sample processing. Complete the missing elements, revise the design, or lower the target claim. If the intended claim changes during the project, amend the protocol beforehand and repeat Step 1; the additional evidence needed for the stronger claim must be collected, not inferred. A discovery study should never be described afterwards as clinically validated. Conversely, clinical validation does not remove the need to keep the raw MS data and the analytical QC record.

*Record.* A version-controlled study plan.

### 3.3. Step 2: Check That Each Specimen Is Fit for Analysis

*Input.* The specimens and their pre-analytical records. The specimen is the first part of the measurement system: its collection, processing, and storage can change the molecular profile before analysis begins, and such changes cannot be corrected later by analytical QC [7,8].

*Action 2.1. Collect the pre-analytical data.* Record identity, anatomical or biofluid source, collection device, additives, collection time, time to processing, centrifugation conditions, temperature history, aliquoting, storage time, and freeze–thaw cycles, to the extent required by the matrix and the intended use [7,8,25,39,69]. The Standard PREanalytical Code (SPREC) version 4.0 offers a structured way to code these variables, and ISO 20387:2018 sets requirements for competence, traceability, and fitness for purpose [25,39]. A revised edition of ISO 20387 reached the final draft stage in 2026 [70]; the edition in force at the time of the study should be applied. The main risks in each omics field are summarised in Table 5.

*Action 2.2. Apply acceptance limits defined in advance.* Typical limits are the maximum time to stabilisation, acceptable temperature excursions, haemolysis or other matrix quality indicators, the maximum number of freeze–thaw cycles, the minimum volume, and mandatory metadata fields. These limits cannot be universal, because stability depends on the analyte and the matrix. Where published data are lacking, test stability in the same matrix, container, storage conditions, and analytical readout as in the study [8,69].

*Action 2.3. Record deviations.* In this protocol, a pre-analytical deviation is any documented difference between the actual handling of a specimen and the conditions in the approved study plan, such as delayed centrifugation, a temperature excursion, or an additional freeze–thaw cycle. This scientific definition differs from a deviation in the regulatory sense of GLP or GMP, where every departure from an approved study plan or standard operating procedure must be formally recorded, assessed, and authorised, whatever its scientific effect [66]. In both settings, a deviation may still be usable if its effect is known and taken into account. Undocumented differences between specimens are a greater problem than a documented and quantified limitation.

*Checkpoint 2.* Classify each specimen as accepted (PASS), accepted with a flag (CONDITIONAL PASS; the deviation is used as a covariate and examined in a sensitivity analysis in Step 5), or rejected (FAIL). Step 2 as a whole is passed only if flagged and rejected specimens are not distributed so unevenly between the groups that they confound the main comparison.

*If the checkpoint is not passed.* Obtain replacement specimens, rebalance the design, or lower the target claim.

*Record.* A list of qualified specimens with flags.

### 3.4. Step 3: Prepare Samples and Acquire Data Under Control

*Input.* The qualified specimens, the QC plan, and a randomised or blocked order for preparation and injection. Sample preparation is part of the measurement procedure, because it decides which fraction of the biological system reaches the detector.

*Action 3.1. Prepare samples with controls suited to the omics field.* In bottom-up proteomics, the usual stages are lysis or extraction, reduction, alkylation, enzymatic digestion, cleanup, and loading normalisation. Digestion can be monitored through missed cleavages, peptide yield, and sequence coverage, and studies of post-translational modifications need controls for the enrichment step [1,56,71]. In untargeted metabolomics, metabolism must be stopped quickly, and the extraction must suit the polarity of the analytes and the matrix. Isotope-labelled internal standards should be added as early as possible, so that they reflect more than injection variability [2,8,74,75]. In lipidomics, record the extraction chemistry, the use of antioxidants, the container material, evaporation and reconstitution, and which internal standard is used for which lipid class. Because ionisation efficiency differs strongly between lipid classes, one internal standard is rarely enough for broad quantitative claims [3,22,76]. For glycomics and glycoproteomics, record the biological source, release method, purification, labelling or derivatisation, enrichment, and any reaction that could remove or rearrange labile structures, following the MIRAGE guidelines [4,23]. In metallomics, the preparation depends on what is to be measured. Complete digestion is suitable for total element content but destroys information on chemical species, whereas speciation methods must keep the chemical form of the element unchanged during extraction, storage, and separation [6,73]. In low-input and single-cell work, losses and contamination become comparable to the endogenous signal. Miniaturisation, automation, low-binding consumables, carrier strategies, and blank controls should therefore be treated as analytical variables in their own right [16,77].

*Action 3.2. Add each control at the right point.* A control can show only the variation that arises in the stages it passes through; it says nothing about stages that took place before it was added. An isotope-labelled peptide added after digestion controls the LC–MS response but not proteolysis. A metabolite internal standard added before extraction also reflects extraction losses. A class-specific lipid internal standard can reveal class-dependent ionisation or recovery. For each control, the QC plan states the material, the point of addition, the frequency, and the property monitored (Table 6).

*Action 3.3. Check system suitability before the study samples.* The test should cover the properties that could invalidate the dataset: mass calibration and mass accuracy, retention and peak shape, source response, sensitivity, carryover, fragmentation, and, where relevant, ion mobility or imaging performance. Calibration with a calibrant solution is essential for establishing and checking mass accuracy, particularly in high-resolution MS, and should be verified before the sequence and, if the platform requires it, during the sequence. A calibrant solution does not pass through sample preparation or chromatography, so it should be supplemented with a characterised digest, a pooled matrix QC, a reference material, or another material that tests the whole analytical path [9,31,46,52].

*Action 3.4. Build the sequence and monitor it.* Include conditioning injections, system suitability tests, solvent and procedural blanks, internal standards, pooled or process QC samples spread over the batch, study samples in randomised or blocked order, and, if available, long-term reference material [9,21]. Monitor ionisation with internal standards and repeated QC injections. Trends in the total ion current are useful as additional information but can hide suppression of individual analytes or lipid classes [3,9,21]. Control charts with warning and action limits based on earlier QC data, similar to the multirule Shewhart charts used in clinical chemistry [57], show trends before limits are exceeded. Software is available for long-term monitoring of proteomic and targeted assays [58,78,79]. Tools such as iDIA-QC, which is based on AI, and PeakQC, which can be used in any omics field, combine several metrics instead of relying on a single threshold [44,45].

*Checkpoint 3.* The sequence fails when a system suitability or in-run QC result falls outside its predefined action limit, or when a trend breaks a predefined control chart rule, for example, several consecutive QC results beyond a warning limit.

*If the checkpoint is not passed.* Stop the sequence and find the cause. In research laboratories, record the date, the observation, the probable cause, the corrective action, and the affected injections. Laboratories working under ISO/IEC 17025, ISO 15189, GLP, or GMP also handle the failure as a formal nonconformity or deviation [26,27,66]. The affected interval lies between the last passing QC injection and the next passing one. Depending on the cause, clean the source, recalibrate, replace the mobile phase or column, repeat the system suitability test, reinject, re-prepare, or exclude samples with a documented reason.

*Record.* Raw data and a run QC record that links each file to its position in the sequence and to the QC results around it.

### 3.5. Step 4: Check the Dataset Before Release

*Input.* Raw vendor files with sample identifiers, sequence position, acquisition method, and links to QC results; the processing record, including software and version, database or spectral library with version and download date, search or feature detection parameters, and alignment rules; and the QC metrics. Keep the original files when converting formats. The mzQC format and the spectral library and annotation formats mzSpecLib and mzPAF make these records easier to exchange between laboratories [24,80].

**Figure 3:**
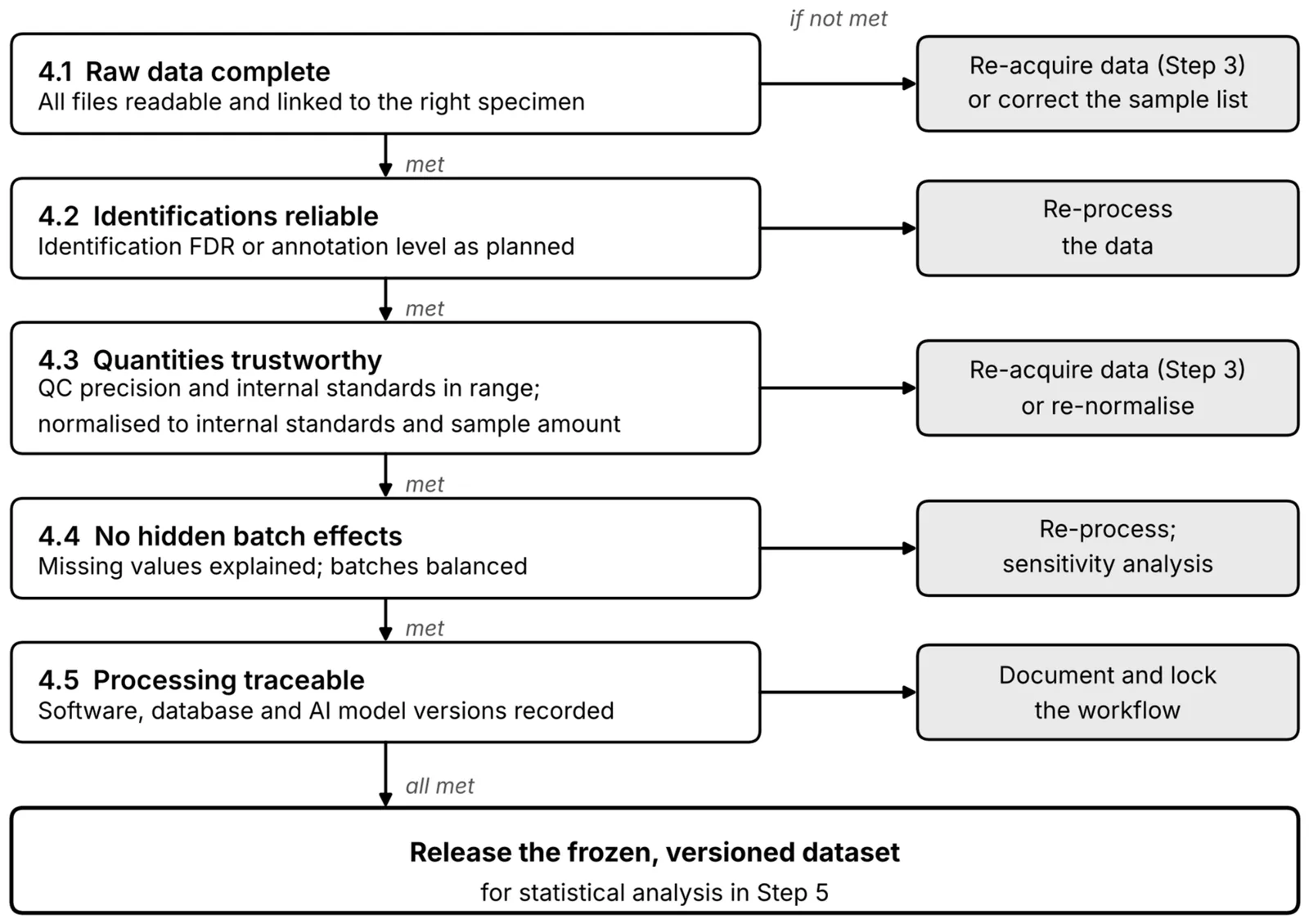
The five checks of Step 4, completed before statistical analysis. Check 4.1: all raw files are complete, readable, and correctly assigned to their specimens. Check 4.2: identifications meet the planned identification FDR or annotation level. Check 4.3: quantitative QC (for example, pooled QC precision and internal standard response) is within range, and the data are normalised to internal standards and to sample amount (for example, tissue mass, protein content, cell number, or urinary creatinine or osmolality). Check 4.4: missing values are explained, and batch effects are absent or corrected. Check 4.5: software, database, and AI model versions and parameters are recorded. A failure that arises in data generation sends the affected interval back to Step 3 (reacquisition or re-preparation); a failure in data processing is corrected by documented reprocessing of the stored raw data. Only a dataset that passes all five checks is frozen, given a version number, and released for statistical analysis in Step 5. AI, artificial intelligence; FDR, false discovery rate; QC, quality control.

*Actions.* Complete the five checks shown in Figure 3 in the order given. Starting values for the checks are listed in Table 7.

*Check 4.1. Raw data.* All files are complete and readable, each file is assigned to the correct specimen and sequence position, and the acquisition metadata are kept.

*Check 4.2. Identification.* Express identification confidence in the terms used in each field. In proteomics, target–decoy methods control the identification false discovery rate (FDR) at the level of peptide–spectrum matches (PSMs), peptides, and proteins; report each controlled level separately [32,53]. This identification FDR, the expected proportion of wrong identifications in a list, is different from the statistical FDR used in Step 5 for the proportion of false positives among features called differentially abundant, for example with the Benjamini–Hochberg procedure [81]. The two are estimated separately, and control of one does not imply control of the other. In metabolomics, the MSI levels describe identification confidence. A Level 1 identification requires comparison with an authentic standard measured under the same conditions using at least two independent, orthogonal properties [20], and more detailed confidence scales have been proposed for high-resolution MS [82]. Lipid annotations should report only the structural level supported by the data, without assigning double-bond positions or stereochemistry that were not measured [3,22,72]. Glycan and glycopeptide assignments follow MIRAGE [4]. In metallomics, total element content, operationally defined fractions, identified species, and spatial signals must be kept apart [73]. Widely used software, for example MaxQuant [83], MSFragger [84], DIA-NN [14], Skyline [85], and Perseus [86] in proteomics, or XCMS [87], MZmine [88], and MS-DIAL [89] in metabolomics and lipidomics, does not by itself guarantee valid results. The same raw data can give different feature lists or conclusions when the database, peak picking, alignment, FDR, annotation, normalisation, or imputation settings change [12,47].

*Check 4.3. Quantitative QC and normalisation.* Before normalisation or statistical testing, check the internal standard response, precision and drift in pooled QC samples, agreement between replicates, mass and retention time distributions, blank contributions, signal range, and feature detection. Then normalise the data at two levels. Technical normalisation corrects for variation in extraction, injection, and ionisation, preferably with internal standards matched to the analytes or analyte classes; where these are not available, QC-based drift correction or data-driven methods can be used [9,60,61]. Normalisation to sample amount corrects for differences in starting material, using a separately measured quantity such as tissue mass, protein content, cell number, or DNA content, or creatinine or osmolality for urine [60]. Choose the normalisation method in advance and check its effect. Normalisation can reduce a systematic technical effect, but it must not be used to hide a failed run. Drift correction needs enough QC injections across the sequence, and nonlinear correction is particularly unreliable when QC injections are sparse [9,21,74,75].

*Check 4.4. Missing values and batches.* Decide first whether missing values reflect a measurement problem, censoring of low-abundance signals, random acquisition effects, or true biological absence. Imputation without this assessment can change effect sizes and the ranking of features. Compare the conclusions under reasonable alternative treatments in a sensitivity analysis, keep the original pattern of missing values, and report the proportion of imputed values [47,51,59]. Use principal component analysis, clustering, and correlation plots to detect drift, outliers, and batch structure; batch effects must be absent or corrected before biological testing [10]. Do not remove a sample that looks like an outlier without an analytical or biological reason documented from the raw data and metadata.

*Check 4.5. Traceability of data processing.* Record the versions of software, databases, and models, together with parameters and exclusions. For processing steps that use AI, also record the model version (including the saved model weights), the data used for training or fine-tuning, the input preprocessing, and the confidence output, and keep the settings needed to reproduce stochastic models. When predictive claims are made, lock the model before evaluating it on an independent validation set, and treat any later change as a new version of the pipeline [18,62,90,91]. AI output does not replace conventional QC or independent chemical evidence. Multimetric AI models such as iDIA-QC still rest on interpretable QC metrics and on performance states labelled by experts [44], and a high score for a predicted spectrum or retention time does not by itself prove chemical identity [18,90].

*Checkpoint 4.* Release the dataset only when all five checks pass. A CONDITIONAL PASS is possible when a limited problem remains, for example, a smaller proportion of features passing the precision filter than planned, provided that the problem is reported and the claim level is adjusted.

*If the checkpoint is not passed.* Failures that arise in data generation (Checks 4.1 and 4.3, for example, corrupted files or loss of sensitivity) send the affected interval back to Step 3 for reacquisition or re-preparation. Failures that arise in data processing (Checks 4.2–4.5, for example, an unsuitable database or normalisation setting) are corrected by documented reprocessing of the stored raw data.

*Record.* A frozen dataset with a version number and checksums, and its release record.

### 3.6. Step 5: Analyse and Interpret the Data

*Input.* The released dataset, the statistical analysis plan, and, where used, locked AI models. The plan defines transformations, normalisation, covariates, the structure of repeated measures, prespecified contrasts, correction for multiple testing, reporting of effect sizes, exclusion of samples or features, and the validation strategy [48,56,67].

*Action 5.1. Follow the plan.* Document and justify every deviation from it.

*Action 5.2. Report effects with their uncertainty.* Give the direction and size of each change together with its uncertainty and the significance adjusted for multiple testing [81]. Keep technical and biological variance separate [48]. Large fold changes with poor analytical reproducibility call for caution, whereas smaller but consistent effects may be biologically important even when an underpowered pilot study does not reach conventional significance.

*Action 5.3. Prevent data leakage in predictive models.* Select features and tune hyperparameters only inside the training folds. Group cross-validation by independent biological specimen and, where appropriate, by site or batch. Assess the final performance in independent material that was not used for feature discovery, threshold setting, or tuning [62,63,64].

*Action 5.4. Integrate omics layers only after each layer has passed Step 4.* Feature-level, latent factor, pathway or network, and decision-level methods answer different questions. MOFA+, DIABLO, and mixOmics are examples of integration frameworks, but no algorithm can restore analytical validity that the individual datasets lack [92,93,94]. Check sample correspondence, identifiers, scaling, missing modalities, and batch alignment before integration. Disagreement between layers is not automatically a QC failure. Transcript abundance may not predict protein abundance, protein abundance may not determine metabolic flux, and element concentration may not identify the active species. Pathway enrichment and interaction networks remain hypothesis-generating unless supported by consistent measurements and independent evidence, and the background set should contain the features that the method could actually detect [95].

*Action 5.5. Run sensitivity analyses.* Test whether the conclusions hold when flagged specimens (Step 2) are excluded and when other reasonable imputation and normalisation choices (Step 4) are used.

*Checkpoint 5.* Move on when the analysis followed the plan, multiple testing was controlled, no leakage occurred, and the sensitivity analyses agree. Assign the claim level reached according to Table 3.

*If the checkpoint is not passed.* Reanalyse according to the plan or lower the claim. For example, report an intended mechanistic (C2) claim as an exploratory (C1) association. Do not change the analysis until the target level is reached.

*Record.* An interpretation report that states the claim level reached and all deviations from the plan.

### 3.7. Step 6: Verify the Findings and Report

*Input.* The candidate findings with their claim level, and the independent material, orthogonal method, or targeted assay required by the target level.

*Action 6.1. Choose verification that matches the target claim.* Repeated measurement of the same extract shows only part of the technical repeatability. Re-preparation of the same specimen tests more of the analytical process. Independent biological material tests whether the finding can be generalised. Targeted MS, authentic or isotope-labelled standards, orthogonal techniques, immunoassays, and functional experiments provide different and complementary evidence [20,50,56]. For targeted peptide and protein assays, address selectivity, calibration and response, repeatability, intermediate precision, matrix effects, recovery, carryover, stability, working range, and robustness, as far as the intended use requires [50,96]. For lipids and glycans, do not report more structural detail than chromatography, fragmentation, and standards support [4,22,76].

*Action 6.2. Report the study and deposit the data.* Combine the record of the six checkpoints with the reporting standards of each field: MIAPE and the Human Proteome Project guidelines for proteomics [19,53], the mQACC recommendations for QA/QC in metabolomics [21], the Lipidomics Reporting Checklist [22,76], MIRAGE for glycoscience [4], and mzQC for QC metrics [24], in line with the FAIR principles [49]. Where ethical, legal, and contractual conditions allow, deposit raw and processed data in field-specific repositories: ProteomeXchange for proteomics, which in 2026 extended its support for FAIR raw data, processed results, and metadata [65]; MetaboLights and the Metabolomics Workbench for metabolomics and lipidomics [97,98]; and GlycoPOST for glycomics MS data [99].

*Action 6.3. Keep the chain of records complete.* Traceability in omics goes beyond metrological calibration. The record should link the specimen identifier with its collection and storage data, preparation batch, raw files, acquisition method, calibration and QC results, file conversions, versions of software, databases, and models, processing parameters, statistical scripts, exclusions, and the final interpretation. Such a computational chain of custody is particularly important when AI models or frequently updated databases are used, because the same raw data can be interpreted differently after a model or library update [18,24,44,90,91].

*Checkpoint 6.* Report the finding at the target claim level only when the verification evidence matches that level.

*If the checkpoint is not passed.* Report the finding at the lower level that the evidence supports, together with the verification needed to reach the target level.

*Record.* The final report, the repository identifiers, and the complete release record of the study.

## 4. Discussion

### 4.1. What the Protocol Adds to Existing Guidance

The protocol does not introduce a new MS technique or analytical method. It adds a layer of decisions on top of existing guidance. Table 8 compares the main documents in terms of scope and purpose and lists what the protocol adds to each. Most existing documents define what should be reported, or which controls should be used, within one field or at one stage of the work [4,19,20,21,22,24]. The protocol states, for every step, what happens when a criterion is not met and who decides. It also separates failures that require new data from failures that can be corrected by reprocessing or accepted with a restricted conclusion. This distinction is implicit in QC practice [9,31] but has not been set out for all omics fields. The claim levels link the record of the six steps to the strongest conclusion that the data support. In this way, they connect analytical QC with the phase models of biomarker development [30,50,55], a connection that analytical guidance usually does not make. Finally, the same terms for control materials, point of addition, and monitored property are used in five chemically different fields, which helps teams with different backgrounds to agree on release criteria in multi-omics projects.

Four common situations show the practical difference. If pooled QC drift appears late in a sequence, metabolomics guidance describes how to assess and correct it [9,21]. The protocol also decides whether the interval must be reacquired, for example when sensitivity has fallen below the action limit, or may be corrected computationally when the drift is limited and QC injections are frequent enough, and it records what this means for the claim. If some specimens exceed a pre-analytical limit, biobanking standards require the deviation to be documented [25,39]. The protocol decides whether these specimens are excluded or kept as a covariate, and whether the resulting imbalance requires a change in design. If an AI model used for library generation or rescoring is updated during a project, reporting recommendations ask for the version to be disclosed [62]. The protocol treats the update as a new pipeline whose output must pass Step 4 again. If a discovery finding is proposed as a biomarker, the protocol does not allow it to be presented as verified or clinically relevant until Step 6 provides evidence at levels C3–C5.

### 4.2. Implementation in the Laboratory

No new instruments are needed. Steps 1 and 2 are planning and documentation completed before analysis. Step 3 uses controls that most laboratories already run. Step 4 can be largely automated with QC software that evaluates predefined metrics and exports them in mzQC [24,45,78,79]. Steps 5 and 6 make visible decisions that are otherwise often taken informally. The record of the six checkpoints can be kept as templates in an electronic laboratory notebook or a laboratory information management system, using Table 2 as the starting point. A core facility can carry out Steps 2–4 and hand over a released dataset together with its record, while the investigator remains responsible for Steps 1, 5, and 6. Retrospective or public datasets can be audited in the same way. Missing evidence does not necessarily make such data unusable, but it limits the claim they can support.

### 4.3. Use Across Platforms and Omics Fields

The order of the steps is the same for every platform; the criteria within each step are not. The protocol applies to liquid and gas chromatography–MS, capillary electrophoresis–MS, direct infusion and ion mobility MS, MS imaging, and inductively coupled plasma MS, and to data-dependent, data-independent, and targeted acquisition. What changes between platforms are the system suitability test, the QC materials, and the identification framework. Proteomics and untargeted metabolomics have the most developed quantitative QC conventions, which is why most of the starting values in Table 7 come from these fields [9,21,31,32]. Lipidomics has well-developed nomenclature and reporting standards but still faces problems with quantitative harmonisation [3,72,100]. Glycomics and glycoproteomics have detailed reporting requirements, but few agreed quantitative acceptance limits [4,23]. In metallomics, the trueness of total element determinations can be controlled with certified reference materials and well-established elemental calibration, whereas speciation analysis must also show that the chemical species remain stable during preparation and separation [6,73]. MS imaging adds criteria for spatial registration and section quality [17]. Where agreed limits are missing, the protocol gives the decision rule and asks the laboratory to derive its own limits, without borrowing them from better-studied fields.

### 4.4. Interlaboratory Variability and Transfer of Numerical Limits

Interlaboratory studies show that differences between laboratories arise not only from instruments but also from how procedures are applied and how decisions are made. In the Human Proteome Organization (HUPO) test sample study, most laboratories did not at first report all proteins of a defined mixture, and central reanalysis traced the discrepancies mainly to database searching and data curation, not to data acquisition [11]. Studies of repeatability and reproducibility in proteomic identification [41] and multi-site studies of targeted proteomics and of SWATH-MS data-independent acquisition showed that quantification can be reproduced across laboratories when samples, assays, and analysis pipelines are standardised [40,42,96]. In lipidomics, the interlaboratory exercise of the National Institute of Standards and Technology (NIST) based on Standard Reference Material (SRM) 1950 revealed considerable variation between laboratories and also provided consensus values that can serve as a common reference [29,101]. A standardised, kit-based targeted metabolomics platform achieved good reproducibility between laboratories [43], whereas a survey of laboratories performing untargeted metabolomics found very different QA/QC practices [28].

Two practical consequences follow. First, a numerical limit has meaning only together with its matrix, platform, acquisition mode, processing workflow, and quantitative level, and a limit established under one set of conditions cannot be assumed to hold under another. Values from the literature are useful starting points but should not be treated as universally valid. In untargeted metabolomics, frequently cited examples are a pooled QC feature RSD below 20% (up to 30% for some matrices), a D-ratio below 50%, a detection rate above 70%, and one pooled QC injection every 5–10 study samples [9]. mQACC guidance stresses that the final QC frequency and acceptance rules should be justified by sequence length, technical variability, sample type, and intended use [21,74,75]. Literature values should therefore be used during method development and replaced by control limits derived from the laboratory’s own QC history once enough data are available [57]. Second, in multi-site studies, the criteria, limits, and corrective actions of each step should be agreed and documented before analysis, and shared reference materials and metrics encoded in mzQC should be used so that release decisions are comparable between sites [24,29].

### 4.5. Practical Difficulties

Several difficulties can be expected. Documentation, additional QC injections, and independent review take instrument time and staff effort, which can be a real burden for small laboratories. The protocol reduces this burden by scaling the requirements to the target claim level, so that an exploratory C1 study needs much less verification than a C4 or C5 study. For some fields and matrices, no literature values exist, and a laboratory must first build up a QC history before it can set control limits. Until then, decisions rest on method development data and should be marked as provisional. A CONDITIONAL PASS requires judgement about whether a limitation is really limited. Listing examples of acceptable conditions in the study plan and involving an independent reviewer make this judgement less subjective. When specimens, measurements, and data processing are handled by different institutions, responsibility for each step and the transfer of records must be agreed in advance, for example in collaboration agreements or data management plans. Retrospective datasets often lack pre-analytical data or processing records. The protocol does not reject such data, but it limits the conclusions they can support.

### 4.6. Limitations

The protocol has several limitations. A common framework has to simplify across fields that differ in chemistry, in what is measured, and in how mature their quality conventions are. The order of the steps is shared, but the criteria for glycomics, metallomics, and MS imaging rest on fewer agreed quantitative conventions than those for proteomics and metabolomics. The protocol is based on a narrative review of literature and standards and on our own experience. It has not yet been tested in a prospective multicentre study or through a formal consensus process, so its effect on reproducibility remains to be measured. The numerical values come from the literature and carry with them the matrices, platforms, and purposes of the original studies. AI tools change faster than reporting standards. Model updates, a mismatch between training data and the study, limited interpretability, and data leakage can change results without any change in the specimen or the instrument [18,63,91]. The traceability requirements of Step 4 and model locking reduce these risks but do not remove them, and they will need revision as community standards for AI in MS develop [62,64]. Finally, the protocol does not replace the validation of individual assays. Such validation remains necessary whenever measured concentrations are used for decisions: in regulated bioanalysis, where ICH M10 applies [38]; in clinical LC–MS examinations, which must be validated within an ISO 15189 quality system and, for in vitro diagnostic devices in the European Union, comply with Regulation (EU) 2017/746 [27,102]; when a method is transferred to a new matrix, platform, or laboratory; and when a targeted assay is intended for clinical or other high-tier use [50]. In these cases, the protocol shows when validation is needed and how its result affects the claim, while the validation experiments themselves follow the relevant standards and guidelines [37].

## 5. Conclusions

The protocol presented here is a step-by-step guide for MS-based omics, not a new analytical method. Its new element is the arrangement of existing field-specific guidance and international standards into six steps. Each step has a defined input, actions, a checkpoint with criteria set in advance, corrective actions, and a record, and the claim levels link the collected evidence to the strongest conclusion it supports. The protocol is most useful in multidisciplinary and multi-omics projects in which specimen handling, measurement, data processing, and interpretation are shared between teams, in core facilities that release datasets to investigators, and in studies that move from discovery towards verification. Its main limitations are that the criteria remain specific to each field and laboratory, that the numerical values are starting points and not universal limits, that the protocol has not yet been tested prospectively in several laboratories, and that it complements, but does not replace, the validation of individual assays for clinical or regulatory use.

## Figures and Tables

**Table 1:** Origin of the protocol components. Adopted: taken from existing guidance without substantive change. Adapted: generalised or modified for use across omics fields. New: proposed in this protocol.

Protocol Component	Origin	Based on
Six steps, each ending with a documented checkpoint (input, actions, criterion, corrective action, record)	New	Stage-gate model [54]; validity of results and control of nonconforming work [26,27]
Three possible decisions: pass, conditional pass with a restricted claim, fail	New	Proposed here
Correction according to where the failure arose: reacquire, re-prepare, reprocess, or restrict the claim	New	QC investigation practice [9,21,31]
Claim levels C1–C5	Adapted	Phases of biomarker development [30,55]; tiers of targeted assays [50]; identification levels [20]
Quality requirements for each quantitative level	Adapted	Metabolomics QC guidance [9,21]; targeted MS and bioanalytical guidance [38,50]; lipidomics [3]
Adding each control before the stage it monitors	Adapted	Metabolomics and proteomics QC guidance [9,21,31,56]
Pre-analytical data and specimen acceptance	Adopted	[7,25,39]
System suitability, pooled QC, blanks, and sequence design	Adopted	[9,21,31,46,52]
Control charts with warning and action limits	Adopted	[57,58]
Numerical starting values	Adopted; labelled as starting values	[9,21,32,38,53]
Classification of missing values and sensitivity analysis	Adapted	[47,51,59]
Normalisation at two levels: technical and sample amount	Adapted	[60,61]
Traceability of AI models, model locking, and prevention of data leakage	Adapted	[62,63,64]
Reporting and data deposition	Adopted	[4,19,21,22,24,49,65]

AI, artificial intelligence; QC, quality control.

**Table 2:** The six steps at a glance. Read each row from left to right: what to do, when to move on, what to do if the checkpoint is not passed, and what to record.

Step	What to Do	Move On Only If	If Not	Record
**1. Define the intended use and the target claim**	Write the intended-purpose statement; choose the claim level and the quantitative level; plan the design, statistics, and QC	The plan is complete and approved before any sample is processed	Revise the plan or lower the target claim	Approved study plan with version number
**2. Check that each specimen is fit for analysis**	Collect pre-analytical data (e.g., SPREC); apply acceptance limits; record deviations	Each specimen is accepted, flagged, or rejected, and the groups remain balanced	Replace specimens, rebalance the design, or lower the claim	List of qualified specimens with flags
**3. Prepare samples and acquire data under control**	Add controls at the right point; test system suitability; monitor QC during the run	System suitability passes before the samples; QC stays within action limits	Stop, correct the cause, reinject or re-prepare the affected interval	Raw data and run QC record
**4. Check the dataset before release**	Complete five checks: raw data, identification, quantitative QC and normalisation, missing values and batches, traceability	All five checks pass	New data (back to Step 3) or documented reprocessing	Frozen dataset with version number
**5. Analyse and interpret the data**	Follow the analysis plan; control multiple testing; prevent data leakage; integrate omics layers	The analysis followed the plan and the sensitivity analyses agree	Reanalyse as planned or lower the claim	Report with the claim level reached
**6. Verify the findings and report**	Confirm findings in independent material or with an orthogonal method; report; deposit data	The evidence matches the target claim level	Report at the lower level; plan verification	Final report, repository identifiers, release record

QC, quality control; SPREC, Standard PREanalytical Code.

**Table 3:** Claim levels: the evidence needed before each type of conclusion can be reported.

Claim Level	Typical Study	Evidence Needed	Not Yet Justified
**C1 Exploratory association**	Discovery	Steps 1–5 passed; representative cohort; balanced batches; FDR control; transparent feature filtering	Identity beyond the reported confidence level; causality; generalisation
**C2 Mechanistically supported association**	Mechanistic	C1 plus a prespecified hypothesis, a controlled intervention, and independent biological evidence	Analytical specificity of single features; clinical use
**C3 Analytically verified finding**	Targeted verification	C1 or C2 plus targeted or orthogonal measurement with authentic or isotope-labelled standards; precision, selectivity, carryover, and stability tested	Generalisation beyond the discovery samples
**C4 Independently replicated finding**	Independent replication	C3 plus new specimens or an independent cohort studied under a protocol set in advance	Diagnostic or prognostic performance, unless the study was designed for it
**C5 Clinically validated result**	Translational or clinical	C4 plus clinical validation in the intended population; validated examination in a medical laboratory (ISO 15189)	Use outside the validated purpose

FDR, false discovery rate.

**Table 4:** Quality requirements for each quantitative level. Values are starting points to be confirmed for the matrix, platform, and intended use.

Requirement	Qualitative	Semi-Quantitative (Relative)	Quantitative (Absolute or Targeted)
Aim	Identify; presence or absence; profiling	Compare relative amounts between groups	Report concentration or amount in defined units
Internal standards	Optional (to follow retention time and injection)	Recommended; added before extraction or digestion; class-matched in lipidomics	Required; a stable-isotope-labelled analogue for each analyte where possible
Calibration	Mass calibration only	Check response linearity, e.g., with a pooled QC dilution series [9]	Matrix-matched calibration with authentic standards over a defined range
QC materials	System suitability material; procedural blanks	As for qualitative studies, plus pooled QC throughout the run and long-term reference QC	As for semi-quantitative studies, plus QC samples at low, medium, and high concentration; reference material if available
Precision (starting value)	Reproducible detection in replicates	Pooled QC feature RSD < 20% (up to 30% for some matrices) [9,21]	CV ≤ 15% (≤20% at the LLOQ) [38]
Accuracy	Not applicable	Not assessed in absolute terms; direction and size of change only	Within ±15% of the nominal value (±20% at the LLOQ) [38]
Identification	FDR or MSI level reported	As for qualitative studies	Authentic standard (MSI Level 1); analyte-specific transitions or fragments
Claim levels usually reached	C1	C1–C2; C3 after orthogonal verification	C3–C5

CV, coefficient of variation; FDR, false discovery rate; LLOQ, lower limit of quantification; MSI, Metabolomics Standards Initiative; QC, quality control; RSD, relative standard deviation.

**Table 5:** Main risks in each omics field, how to control them, and what to check in Steps 3 and 4.

Omics Field	Main Risks	What to Do	What to Check in Steps 3 and 4
**Proteomics, including PTM analysis**	Proteolysis; dephosphorylation; incomplete digestion; adsorption at low input	Stabilise quickly; use inhibitors where needed; add process QC before lysis; use low-binding consumables	Missed cleavages; QC peptide intensity; identification FDR; retention time and mass error [1,31,56,71]
**Metabolomics**	Ongoing metabolism; effects of tubes and additives; time and temperature before processing	Quench or separate quickly; add internal standards early; include pooled QC and blanks	Pooled QC RSD; D-ratio; internal standard response; blank contribution [2,8,9,21]
**Lipidomics**	Oxidation and hydrolysis; class-dependent extraction; isomers	Limit exposure to air, light, and heat; use class-specific labelled standards	Recovery of class-specific internal standards; pooled QC RSD; structural level reported [3,22,72]
**Glycomics and glycoproteomics**	Incomplete release; loss of labile residues; variable derivatisation	Document release, purification, and derivatisation; use process standards	MIRAGE metadata; fragment and retention time evidence for isomers [4,23]
**Metallomics and speciation**	Contamination; adsorption; conversion between species	Use trace-metal-clean materials, procedural blanks, and CRMs; keep pH and redox conditions stable	Blank contribution; recovery for the CRM; stability of species [6,73]

CRM, certified reference material; FDR, false discovery rate; PTM, post-translational modification; QC, quality control; RSD, relative standard deviation.

**Table 6:** Control materials: when to add them, what they show, and what they cannot show.

Control	When to Add It	What It Shows	What It Cannot Show
**Biological replicate**	Independent biological unit	Biological variance; whether the finding generalises	Analytical error
**Technical replicate**	Repeat of a stated stage (injection or preparation)	Variance of that stage	Biological variance; variance of earlier stages
**Procedural blank**	At the start of preparation, without biological material	Contamination from reagents and consumables; carryover	Matrix effects; recovery
**System suitability material**	Before the study samples and at set intervals	Mass accuracy, retention time, sensitivity, peak shape	Variation in sample preparation
**Pooled study QC**	Made from the study specimens and processed with them	Precision and drift within the study; basis for drift correction	Absolute accuracy
**Process QC or long-term reference material**	Before the first preparation stage to be monitored	Recovery; comparability between batches and studies	Matrix effects specific to the study, if not matrix-matched
**Internal standard**	As early as useful, ideally before extraction or digestion	Recovery, matrix effects, and injection or ionisation variation for matched analytes	Variation in analytes or classes it does not match

QC, quality control.

**Table 7:** Starting values for the checks of Step 4. These values support decisions but are not universal limits; each must be justified for the matrix, platform, study design, and intended use.

Check	Starting Value	Notes and Source
Identification FDR (proteomics)	1% at each controlled level (PSM, peptide, protein), reported separately	Common in discovery work; must suit the claim [32,53]
Pooled QC feature RSD (untargeted)	<20%; <30% for some matrices or workflows	Community guidance; report how many features are kept and removed [9,21]
D-ratio (untargeted)	<50%, preferably much lower	Technical versus biological spread; use together with precision and detection [9]
Detection rate in pooled QC	>70%	Example from community guidance, not a fixed rule [9]
Pooled QC frequency	Every 5–10 study samples, plus conditioning and closing QC injections	Adapt to batch length, variability, and planned drift correction [9,21]
Mass accuracy, retention time, peak shape, internal standard response	Warning and action limits from the laboratory’s own QC data; no universal value	Derived from QC history and control charts [31,46,52,57]
Blank contribution	Sample-to-blank ratio set in advance	Depends on the workflow; set before unblinded analysis [9,21]
Targeted quantitative assays	CV ≤ 15% (≤20% at the LLOQ); bias within ±15% (±20% at the LLOQ)	Regulated bioanalysis; fit-for-purpose tiers for research assays [38,50]

CV, coefficient of variation; FDR, false discovery rate; LLOQ, lower limit of quantification; PSM, peptide–spectrum match; QC, quality control; RSD, relative standard deviation.

**Table 8:** How the protocol relates to existing guidance and standards.

Document	Field	Main Purpose	What the Protocol Adds
**MIAPE** [19]	Proteomics	Minimum reporting	Acceptance criteria before release; what to do after a failure
**MSI identification levels** [20]	Metabolomics	Identification confidence	Link between identification level and permitted claim
**Metabolomics QC guidance; mQACC** [9,21]	Untargeted metabolomics	QA/QC practice and reporting	The same decision rules for other fields; claim levels
**Lipidomics Standards Initiative guidance and checklist** [3,22]	Lipidomics	Good practice and reporting	Acceptance and escalation rules for each step
**MIRAGE** [4,23]	Glycomics and glycoproteomics	Reporting of sample preparation and MS	Decision rules where quantitative conventions are missing
**mzQC** [24]	All MS fields	Format for QC metrics	Which metrics are checked at which step, and with what consequence
**ISO 20387** [25]**; SPREC** [39]	Biobanking	Competence, traceability, pre-analytical coding	Decision on specimen acceptance (Step 2)
**ISO/IEC 17025** [26]	Testing laboratories	Competence; validity of results	Omics-specific criteria within the quality system (Steps 3 and 4)
**ISO 15189** [27]	Medical laboratories	Quality and competence of examinations	Boundary between research claims (C1–C4) and clinical use (C5)

MIAPE, Minimum Information About a Proteomics Experiment; MIRAGE, Minimum Information Required for a Glycomics Experiment; mQACC, Metabolomics Quality Assurance and Quality Control Consortium; MSI, Metabolomics Standards Initiative; SPREC, Standard PREanalytical Code.

## Data Availability

No new data were created or analysed in this study. Data sharing is therefore not applicable. All sources used to develop the proposed methodological framework are cited in the article.

## Abbreviations

The following abbreviations are used in this manuscript:

AI	Artificial intelligence
CRM	Certified reference material
CV	Coefficient of variation
DIA	Data-independent acquisition
FAIR	Findable, Accessible, Interoperable, and Reusable
FDR	False discovery rate
GLP	Good laboratory practice
GMP	Good manufacturing practice
HRMS	High-resolution mass spectrometry
HUPO	Human Proteome Organization
ICH	International Council for Harmonisation of Technical Requirements for Pharmaceuticals for Human Use
IEC	International Electrotechnical Commission
ISBER	International Society for Biological and Environmental Repositories
ISO	International Organization for Standardization
LC–MS	Liquid chromatography–mass spectrometry
LLOQ	Lower limit of quantification
MIAPE	Minimum Information About a Proteomics Experiment
MIRAGE	Minimum Information Required for a Glycomics Experiment
mQACC	Metabolomics Quality Assurance and Quality Control Consortium
MS	Mass spectrometry
MSI	Metabolomics Standards Initiative
NIST	National Institute of Standards and Technology
PSM	Peptide–spectrum match
PTM	Post-translational modification
QA	Quality assurance
QC	Quality control
RSD	Relative standard deviation
SPREC	Standard PREanalytical Code
SRM	Standard Reference Material
